# CCL5/RANTES Gene Polymorphisms in Slavonic Patients with Myocardial Infarction

**DOI:** 10.1155/2011/525691

**Published:** 2011-03-29

**Authors:** Irina P. Tereshchenko, Jana Petrkova, Mikhail I. Voevoda, Milos Taborsky, Zdenka Navratilova, Aida G. Romaschenko, Vladimir N. Maksimov, Frantisek Mrazek, Martin Petrek

**Affiliations:** ^1^Laboratory of Immunogenomics and Proteomics, Institute of Molecular and Translational Medicine, Palacky University and Faculty Hospital, 775 20 Olomouc, Czech Republic; ^2^Institute of Internal Medicine, Siberian Branch of Russian Academy of Medical Sciences, Novosibirsk 630089, Russia; ^3^Internal Medicine—Cardiology, Palacky University and Faculty Hospital, 775 20 Olomouc, Czech Republic; ^4^Cardiogenetics and Immunogenetics Laboratory, Palacky University and Faculty Hospital, 775 20 Olomouc, Czech Republic

## Abstract

Coronary artery inflammation is a critical process in the pathogenesis of myocardial infarction (MI). The chemokine CCL5/RANTES (regulated upon activation, normal T cells expressed and secreted) is expressed in advanced atherosclerotic lesions. Functional polymorphisms of the RANTES gene can, therefore, be involved in the pathogenesis of coronary artery disease. We examined the association of polymorphisms in the RANTES gene with myocardial infarction in Slavonic populations of Czech and Russian origin. A total of 467 post-MI patients and 337 control subjects were genotyped for RANTES promoter G-403A (rs2107538) and intron 1.1 T/C (rs2280789) variants by PCR-SSP. Both RANTES genotypes and allele frequencies did not differ between case and control groups. Haplotype-based analysis also failed to reveal an association between MI and investigated markers. Strong linkage disequilibrium was detected between particular RANTES alleles. The data do not support an association between RANTES G-403A polymorphism and MI, as reported previously.

## 1. Introduction

Atherosclerosis is a pathological process that takes place in the major arteries and represents one of the causes of myocardial infarction (MI), ischemic stroke, and peripheral artery disease. Atherosclerosis, a multifactorial condition determined by environmental as well as genetic factors, has a strong inflammatory component [1] characterised by deregulation of gene expression in macrophages within atherosclerotic plaques, together with local overproduction of cytokines. Among those, chemokines (chemotactic cytokines) have also been implicated in atherosclerosis on the basis of clinical studies and animal disease models [2]. 

The chemokine RANTES, also known as CC chemokine ligand (CCL5), is involved in the activation and proliferation of T-lymphocytes and is considered as a major chemokine implicated in both the acute and chronic phases of inflammation. Tissue RANTES expression is differentially regulated: in atherosclerosis, RANTES mRNA has been detected in the lymphocytes, macrophages, myofibroblasts, and endothelial cells of human arteries undergoing accelerated atherosclerosis but not in normal coronary arteries [3]. RANTES was also found deposited on activated endothelium or murine atherosclerotic carotid arteries [4]. Platelets, which are also implicated in the atherosclerotic process including myocardial infarction (MI), are known to store RANTES in intracellular granules in the basal state and to secrete it when stimulated with thrombin [5]. 

Single nucleotide polymorphisms in the RANTES gene promoter and intronic region have been described: C-28G, G-403A (relative to transcription start site), polymorphism in intron 1 (In1.1 T/C), and in the 3′untranslated regions [6, 7]. The case-control study investigating RANTES SNP G-403A in Germans associated this SNP with a susceptibility to coronary atherosclerosis [8]; in another study, in Hungarians, however, this association was not significant [9]. That RANTES polymorphisms may play a role in cardiac pathology was also suggested by a report enrolling patients with type 2 diabetes mellitus and end stage renal disease [10]: the carriage of either RANTES -403 G/A or intronic polymorphism (In1.1 T/C) was associated with all-cause mortality due mainly to cardiac events.

Concerning the effect of these SNPs on RANTES expression, an increased gene expression was linked with the minor allele in the RANTES gene at the nucleotide position -403 and -28 [11, 12]. However, *in vitro* functional data are more important for RANTES intronic polymorphism T/C: In1.1C allele or haplotypes that include this variant showed a strong dominant association with rapid progression to AIDS [7]. Furthermore, RANTES gene polymorphisms were associated with the outcomes of HCV and HBV infections [13, 14]. By analogy, atherosclerotic lesions, the underlying cause of coronary artery disease (CAD) and MI, represent a series of highly specific cellular and molecular events of the inflammatory response [1]. Despite convincing evidence for a role of the RANTES polymorphisms in different inflammatory diseases, their role in CAD requires further investigation. 

Given the inflammatory component of coronary atherosclerosis and the lack of case-control investigations of more than one RANTES polymorphism in MI, this study examined the relationship between two RANTES polymorphisms (promoter G-403A, intron1.1 T/C) with their haplotypes and myocardial infarction. In agreement with genetic epidemiology principles, our “replication” study comprised two population cohorts (Czech, Russian) differing in ethnicity from those populations in which the RANTES gene was investigated previously (Hungarian, German).

## 2. Materials and Methods

### 2.1. Study Population

A total of 804 unrelated Caucasian individuals were enrolled into this case-control study, of which 467 were MI cases and 337 healthy controls subjects. Two population groups were recruited: (1) Czech (Olomouc) with 223 MI patients and 140 control subjects and (2) Russian (Novosibirsk) with 244 cases and 197 controls from the MONICA project register.  Table 1 shows demographic and clinical characteristics of the study groups.

 The diagnosis of MI was made according to the criteria recommended by international consensus [15]. MI events were defined on the basis of electrocardiographic (ECG) changes and biomarker (troponin, alternatively creatine-kinase) measurements. Local research ethics committees approved the studies and the subjects provided written informed consent for use of samples in these genetic studies and anonymous publication of the summary data.

### 2.2. SNP Genotyping

Genotype analysis for the RANTES SNPs was performed by polymerase chain reaction with sequence-specific primers (PCR-SSP). The investigated SNPs included RANTES promoter G-403A (rs2107538) and RANTES intron 1.1 T/C (rs2280789). The sequences of specific primers were as follows: allele G, forward 5′ ATG GAT GAG GGA AAG GAG G 3′, allele A, forward: 5′ CAT GGA TGA GGG AAA GGA GA 3′, constant reverse: 5′ TTC TCT GCT GAC ATC CTT AG 3′; intron 1.1 T/C; allele T, forward: 5′ CCT GAT CAG TTT TTC TGT CTT T 3′, allele C, forward: 5′ CCT GAT CAG TTT TTC TGT CTT C 3′, constant reverse: 5′ TGT GAG CTA CTC ATG ACT AC 3′. The internal control primers and conditions for PCR were adopted from a “Phototyping” methodology [16].

### 2.3. Statistical Analysis

Calculations were performed using the SPSS v.13.0 (SPSS Inc, Chicago, IL). Categorical data of the study population was reported as frequencies and percentage, the continuous data as means with standard deviation. The distribution of the RANTES genotypes and alleles were analyzed using the Pearson's contingency table *χ* ^2^-test. Odds ratios and 95% confidence interval (CI) were estimated. A multiple variable logistic regression model was used to estimate the association between genotype and disease. Statistical significance was accepted at *P* < .05. Haplotype analysis was performed using an accelerated expectation-maximization algorithm (software ARLEQUIN, version 3.000).

## 3. Results

### 3.1. Distribution of RANTES Polymorphisms in Patients and Control Subjects

First, we evaluated distribution of RANTES polymorphisms in patients and control subjects (for genotype and allele frequencies see Table 2). Out of 467 and 337 individuals in each group, the genotype data for RANTES intron 1 polymorphism was available in 465 patients and 330 controls as 2 patients and 7 control samples failed to amplify; the overall failure rate was only 1.2% (9/804). Both the RANTES -403 and RANTES In1.1 genotypes were distributed as expected from the Hardy-Weinberg equilibrium (*P* > .05). 

In both the Czech and Russian populations, there were no significant differences in the frequencies of genotypes between the MI cases and controls for the two RANTES polymorphisms studied. The distribution of RANTES genotypes was similar also when both populations were evaluated together (Table 2), except that the frequency of CC homozygous genotype in MI patients (2.8%) was higher than observed in controls (0.9%), but this difference did not achieve a statistical significance. 

Further, we carried out standard allelic association analysis: minor allele frequencies for the two polymorphisms were calculated, and the odds ratio and 95% confidence interval for the RANTES -403A and RANTES In1.1C were 0.996 (95% CI 0.740–1.340) and 1.054 (95% CI 0.759–1.464), respectively, (*P* > .05 in both cases). Also, when evaluating carrier status for -403A and In1.1C alleles, there were no significant differences between MI cases and controls (*P* = .97, df = 1 for AA+AG versus GG -403 genotypes; and *P* = .75, for CC+CT versus TT In1.1 genotypes).

### 3.2. Relationship between RANTES Gene Variants, Demographic Factors, and Myocardial Infarction

Next, the patients were divided into subsets according to gender and age at the time of the 1st myocardial infarction, and univariate analyses were performed. There was no difference in genotype distribution when male and female cases were compared with controls of appropriate gender separately (RANTES G-403A: *P* = .863 for males and *P* = .789 for females; RANTES In1.1 T/C: *P* = .148 for males and *P* = .744 for females). No differences in genotype and alleles frequencies (-403A and In1.1C) were observed between the patients stratified according to the age of MI onset (>50 yr and <50 yr) (Table 3).

Subsequently, multivariate logistic regression analysis was performed, adjusting for age and gender. Also, in the logistic regression analysis, no evidence for association was found between the polymorphisms of the RANTES gene and the risk for MI in our study population (Table 4).

### 3.3. Identification and Comparison of RANTES Haplotypes in the Slavonic Population

The frequencies of combined genotypes and haplotypes of the RANTES G-403A/In1.1T/C loci are listed in Table 5. The combined genotypes GGTT and AGTC were the most frequent (case/control group 62.3%/64% and 18%/18.4%, resp.). No statistically significant difference was observed in the RANTES combined genotypes between the cases and controls (*χ* ^2^ = 4.53, df = 7, *P* = .717). However, we detected strong linkage disequilibrium between the two less common alleles for two RANTES SNPs: allele A of the RANTES promoter G-403A and C allele of the RANTES intron 1 T/C (*D*′ = 0.83 for patients with MI and *D*′ = .88 for control group; *P* < 10^−6^ in both cases).

## 4. Discussion

This case-control study aimed at exploring the association between coronary atherosclerosis manifested by myocardial infarction and two functional polymorphisms in the RANTES (CCL5) gene, the product of which is involved in atherosclerotic inflammation. In this two-population replication study, no significant differences in the distribution of RANTES G-403A, and In1.1 T/C single nucleotide polymorphisms were observed between patients with MI and the control groups. Further, when the subjects were stratified according to their gender and age at the first MI episode, no significant relationships were revealed. Thus, our findings do not support the previous report of association between RANTES promoter G-403A polymorphism and coronary artery disease [8]. Importantly, our report represents the first investigation of two linked RANTES polymorphisms and specifically analysis of their haplotypes in the Slavonic population.

Previous studies associating RANTES SNPs with coronary atherosclerosis [8, 9] concentrated on the RANTES promoter polymorphism (G-403A) and did not explore the relationship between cardiovascular symptomatology and further polymorphisms in the RANTES gene, including its intronic variant In1.1T/C. Only Böger et al. [10], who investigated RANTES promoter and intronic polymorphisms in type 2 diabetes/end stage renal disease, reported that patients carrying the RANTES-403A or In1.1C allele had higher all-cause mortality risk, mainly due to cardiac events. We, however, cannot compare their data with our results, because Boger's study did not include a control population (healthy controls) and also estimated the effect of genetic predictors of survival and functional polymorphisms on mortality in another disease condition.

A difference in exact disease phenotypes may be another less prominent, but still relevant factor explaining the discrepancy between our negative data and the first case-control association study on RANTES, which showed a trend indicating a higher risk for coronary artery disease for Hungarian patients with the -403A allele [9]. The population size of their cohort and our Slavonic population was identical; however, the Hungarian study enrolled patients with severe coronary artery disease (CAD) not necessarily presenting with MI. 

The LURIC (the Ludwigshafen risk and cardiovascular health) study was the second to report the association for -403A allele [8]. We speculate that the deviation of distribution of G-403A genotypes from that expected from the Hardy-Weinberg equilibrium in controls of the LURIC study, may contribute to the discrepancy between LURIC and our findings concerning the relationship of the RANTES -403A variant and CAD. Though smaller in number of enrolled subjects, our Czech and Russian groups were strongly compliant with the HWE. 

Strong linkage disequilibrium was observed throughout the RANTES gene, and in particular between the alleles at the four most polymorphic sites, -403, -28, In1.1, and 3′222 [7]. The In 1.1T occurs in combinations with -403G in 77% of genotyped European Americans (5% had In1.1T/-403A haplotype), and allele In1.1C occurred with frequencies of 0.14 in European Americans, 0.20 in African Americans, and 0.34 in Asians. In the present study, we revealed similar allele In1.1C frequencies in our investigated Slavonic populations as in European Americans, and confirmed strong linkage disequilibrium between two less common variants at RANTES -403 and In 1.1 loci. 

The functional effect of particular SNPs in the RANTES gene and the level of its expression and/or secretion which may provide a mechanism explaining the observed association with CAD [8], is still controversial [7, 11, 12, 17, 18]. The -403A allele has been associated with increased transcription of the RANTES gene, and further evidence of the functional importance of this variant is provided by a significant risk of atopic dermatitis [19], atopy, and asthma in adults [20] but without studying the effect of the -28 or In1.1 loci. Other studies showed an upregulatory role for the -28G allele but did not demonstrate upregulation for -403A [12, 18]. Another study demonstrated that RANTES In1.1C alelle was associated with reduced RANTES expression, -28G allele had a moderate role for expression and -403A allele was not regulatory [7]. Discrepant data on correlations between RANTES polymorphisms and level RANTES expression for other common inflammatory and chronic infection diseases support the need for further investigation of the relationship between variability in RANTES gene and its expression. 

In conclusion, in this two-population-based case-control study, no significant difference was detected in the distribution of two RANTES gene polymorphisms between healthy controls and patients with myocardial infarction, indicating the lack of association between coronary atherosclerosis and RANTES single nucleotide polymorphisms. Our results, therefore, do not support a major involvement of RANTES G-403A and In1.1 T/C polymorphisms in genetic predisposition to myocardial infarction.

## Figures and Tables

**Table 1:** Demographic and clinical characteristics of the study groups (MI patients and control subjects).

	Case group RU	Control group RU	Case group CZ	Control group CZ
Number of subjects	244	197	223	140
Caucasians (%)	244 (100%)	197 (100%)	223 (100%)	140 (100%)
Mean age (years)*	56.6 ± 9.7	43.6 ± 7.4	55.0 ± 8.3	35.7 ± 7.4
Gender (males/females)	167/77	87/110	186/37	87/53
Current smokers (%)	88 (97.7%)^a^	123 (63.7%)^b^	64 (31.7%)^c^	n.a.
Total cholesterol (mmol/l)	6.2 ± 1.3	5.2 ± 1.3	5.7 ± 1.1	5.9 ± 1.0
HDL (mmol/l)	1.2 ± 0.3	1.3 ± 0.4	1.1 ± 0.3	1.4 ± 0.4
Triglycerides (mmol/l)	1.7 ± 0.8	1.1 ± 0.7	2.4 ± 1.6	1.9 ± 1.1
LDL (mmol/l)	n.a.	3.1 ± 1.3	3.4 ± 1.0	3.7 ± 0.9

*Mean ± Standard Deviation for continuous variable.

^a^Smoking habit was available in 90 (36.9%) patients.

^b^Smoking habit was available in 193 (98%) subjects.

^c^Smoking habit was available in 202 (91%) subjects.

RU: Russian, CZ: Czech, HDL: high density lipids, LDL: low density lipids, n.a.: not available.

**Table 2:** RANTES promoter and intron polymorphisms: genotype and allele distribution in Slavonic patients with MI and control populations.

RANTES promoter G-403A				RANTES intron 1.1 T/C			
	Case (*n* = 467)	Control (*n* = 337)	*P* value		Case (*n* = 465)	Control (*n* = 330)	*P* value
GG (%)	310 (66.4)	224(66.5)	.709^a^	TT (%)	354 (76.1)	248 (75.2)	.126^a^
GA (%)	135 (28.9)	101 (30)		TC (%)	98 (21.1)	79 (23.9)	
AA (%)	22 (4.7)	12 (3.6)		CC (%)	13 (2.8)	3 (0.9)	
G allele (%)	755 (80.8)	549 (81.5)	.754^b^	T allele (%)	806 (86.7)	575 (87.1)	.791^b^
A allele (%)	179 (19.2)	125 (18.5)		C allele (%)	124 (13.3)	85 (12.9)	

^a^ *P* value from *χ* ^2^test with 2 df.

^b^ *P* value from *χ* ^2^test with 1 df.

**Table 3:** RANTES genotype frequencies in MI patients and cases subdivided according to age at the first MI episode.

RANTES SNPs	RANTES genotypes	≤50 years			>50 years		
		MI (*n* = 159)	control (*n* = 289)	*P* value	MI (*n* = 308)	control (*n* = 47)	*P* value
G-403A(%)	GG	100 (62.9)	189 (65.4)	.87	210 (68.2)	34 (72.3)	.85
	GA	53 (33.3)	90 (31.1)		82 (26.6)	11 (23.4)	
	AA	6 (3.8)	10 (3.5)		16 (5.2)	2 (4.3)	

		MI (*n* = 158)	control (*n* = 284)	*P* value	MI (*n* = 306)	control (*n* = 45)	*P* value

In1.1 T/C(%)	TT	122 (77.2)	215 (75.7)	.21	232 (75.8)	32 (71.1)	.33
	TC	31 (19.6)	66 (23.2)		66 (21.6)	13 (28.9)	
	CC	5 (3.2)	3 (1.1)		8 (2.6)	0	

**Table 4:** Multivariate logistic regression analysis of the MI risk according to RANTES genotypes in the Slavonic population adjusted for age and gender.

Polymorphisms	Genotype	OR	95% CI	*P* value
RANTES -403 G/A	AA versus GA+GG	1.171	0.373–3.673	.79
RANTES In1.1T/C	CC versus TC+TT	2.747	0.451–16.715	.27

**Table 5:** The frequencies of haplotypes (estimated) and combined genotypes of two RANTES single nucleotide polymorphisms G-403A and In 1.1 T/C in the Slavonic population.

HaplotypesG-403A/In1.1 T→C	Case 2*n* = 904	Control 2*n* = 634
AC	0.112	0.114
AT	0.079	0.073
GC	0.017	0.012
GT	0.789	0.800
Combined genotypes		
AATT	0.006	0.054
AATC	0.018	0.017
AGTT	0.126	0.117
AGTC	0.180	0.184
AACC	0.013	0.013
AGCC	0.004	0.003
GGTT	0.623	0.640
GGTC	0.028	0.019
